# Ultrasonic Characteristics of Cardiovascular Changes in Children with Hutchinson–Gilford Progeria Syndrome: A Comparative Study with Normal Children and Aging People

**DOI:** 10.1155/2020/9631851

**Published:** 2020-04-13

**Authors:** Xiao-Ni Zhao, Hong-Ping Song, Fan Yang, Dang-Jie Wu, Wen-Qing Gong, Ying Zhang, Xin Sun, Min-Juan Zheng

**Affiliations:** ^1^Department of Ultrasound, Xijing Hospital, Fourth Military Medical University, Xi'an, 710032 Shaanxi Province, China; ^2^Department of Pediatrics, Xijing Hospital, Fourth Military Medical University, Xi'an, 710032 Shaanxi Province, China

## Abstract

**Background:**

The cardiovascular characteristics of children with Hutchinson-Gilford progeria syndrome (HGPS) remain unclear. The present study is aimed at evaluating the cardiovascular changes with ultrasound examination in children with HGPS and compared these with those in normal children and older people.

**Methods:**

Seven HGPS children, 21 age-matched healthy children, and 14 older healthy volunteers were evaluated by three-dimensional echocardiography (including strain analysis) and carotid elasticity examination with the echo-tracking technique.

**Results:**

Children with HGPS had higher left ventricular ejection fraction (LVEF) and global longitudinal strain, when compared to older healthy volunteers (*P* < 0.05). However, these parameters were not significantly different, when compared to those in healthy children. Furthermore, children with HGPS had lower average peak times in the left ventricle, when compared with the other two groups. For the structure of the carotid artery detected by ultrasound, the abnormality rates were similar between children with HGPS and older healthy volunteers (83.3% *vs.* 71.4%). The elastic parameters, elastic modulus, stiffness parameter, and pulsed wave transmittal velocity of children with HGPS were lower, when compared to those in older healthy volunteers (*P* < 0.05), while they were higher with arterial compliance (*P* > 0.05). Furthermore, no significant difference existed among the vascular elastic parameters between HGPS and normal children.

**Conclusion:**

HGPS children had impaired left ventricular (LV) synchrony, when compared to normal children, although the difference in LVEF was not statistically significant. Furthermore, the structural abnormality of the carotid artery in HGPS children was similar to that in older people, although the index of elasticity appears to be more favorable. These results suggest that the cardiovascular system in HGPS children differs from natural aging.

## 1. Introduction

Hutchinson-Gilford progeria syndrome (HGPS) is an extremely rare sporadic genetic disorder, which is characterized by accelerated pathogenesis of cardiovascular disease and premature aging [1, 2]. Patients with accelerated pathogenesis are generally without significant abnormality in infancy. However, the appearance and symptoms related to aging occur after one year. Previous observations have suggested that children affected by HGPS tend to present with many distinctive features, including alopecia, failure to thrive, short stature, lipodystrophy, joint abnormalities, and facial features resembling elderly people [3, 4]. The prognosis of HGPS is poor, and children with HGPS may suffer from death at a mean age of 13 years, which is probably due to cardiovascular diseases, including myocardial infarction or stroke [3]. However, the vulnerability of patients with HGPS to cardiovascular diseases remains poorly understood, and reports on the cardiovascular feature of patients with HGPS are rare [5], particularly when both structural and functional analyses are performed through ultrasound examinations [6]. Interestingly, a previous study established a mouse model of HGPS and revealed the existence of molecular alterations, which may cause abnormality of cardiac rhythm and premature death in HGPS [7]. Therefore, the systemic evaluation of the structural and functional changes in the cardiovascular system of children with HGPS may be fundamental to understanding the unique pathogenic pattern of cardiovascular death in these patients.

Speckle tracking imaging has been recognized as a useful tool for the angle-independent quantification of myocardial strain [8] and can be applied as a sensitive marker for early myocardial dysfunction before the appearance of left ventricular (LV) remodeling [8, 9]. e-tracking (ET) technology has often been used in predicting artery stiffness, because this can provide real-time pressure-strain elastic parameters [10]. In view of the fact that artery stiffness is an early sign of arteriosclerosis, the early detection of artery stiffness by ET technology may have the potential to predict the risk of arteriosclerosis. With these advanced ultrasound evaluation tools for the cardiovascular system, the present study is aimed at systematically evaluating the structural and functional characteristics of the cardiovascular system in children with HGPS. Specifically, the overall LV function, LV strains, and carotid elasticity were compared among children with HGPS, age-matched healthy children, and older people. The results of the present study may be helpful for understanding the potential vulnerability of children with HGPS to cardiovascular diseases.

## 2. Materials and Methods

### 2.1. Study Population

**Figure 1:**
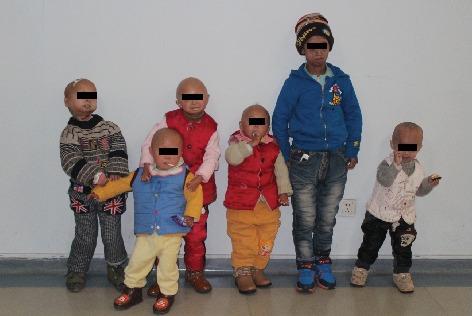
Six children with Hutchinson-Gilford progeria syndrome are presented.

Seven HGPS children diagnosed by genetic analysis in the Pediatric Department of our hospital between 2014 and 2016 were included. One of these children died in 2015 (Figure 1). The baseline characteristics of these included patients at their first admission are presented in Table 1. These patients were scheduled to visit the clinic for four times between 2014 and 2016, in order to obtain the ultrasound data. A total of 21 sets of ultrasound data were obtained after excluding the visits with missing data. Age-matched healthy children (*n* = 21; 13 males and 8 females, age: 22 months old to 9 years old, and mean age: 6.0 ± 2.0 years) and older healthy volunteers (*n* = 14; 6 males and 8 females, age: 52-79 years, and mean age: 65.7 ± 7.5 years) were included as controls. Older healthy volunteers were excluded based on the following criteria: the presence of wall motion abnormalities, LV ejection fraction of <50%, history of cardiomyopathy, vascular heart disease, congenital heart disease, or history of percutaneous coronary intervention (PCI). A written informed consent and assent were obtained from the guardians of the children and the adult participants. The present study was approved by the Ethics Committee of Xijing Hospital (KY20162034-1) before it was conducted.

### 2.2. Echocardiographic Evaluation

**Figure 2:**
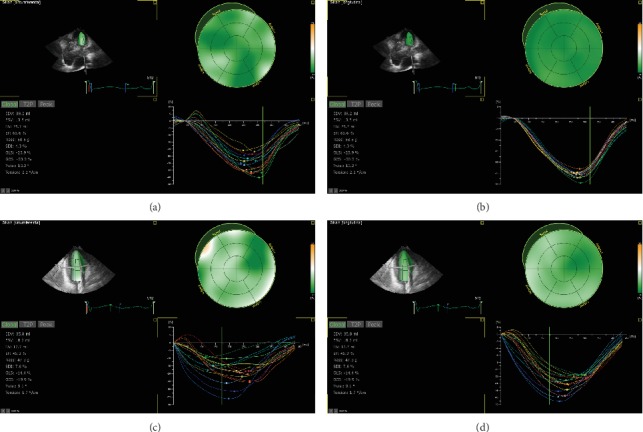
The strain analysis of LV for HGPS and age-matched healthy children. (a) The global circumferential strain (GCS) of age-matched healthy children. (b) The LV global longitudinal strain (GLS) of age-matched healthy children. (c) The GCS of an elder HGPS patient (16 years old). (d) The GLS of an elder HGPS patient (16 years old). A decrease in GCS and GLS was observed in HGPS children.

IE33 (Philips Medical Systems, Andover, MA) echocardiographic machines equipped with an X3-1 transducer (1-3 MHz) were used. Good-quality echocardiographic images were obtained and stored for subsequent offline analyses using the TOMTEC software (TOMTEC Inc., Germany). This was performed by a single investigator, who was blinded to the baseline data of the participants. The 3DE data were first cropped to create orthogonal views equivalent to the apical 4- and 2-chamber views. Then, manual tracing or semiautomated endocardial border tracing techniques were applied, which displayed the time-volume data of the entire LV, as well as the 16 standard myocardial segments, as defined by the American Society of Echocardiography (ASE) [11]. The LV global longitudinal strain (GLS), global circumferential strain (GCS), twist (°), torsion (°/cm), mean peak time (ms), and systolic dyssynchrony index (SDI) were also derived (Figure 2).

### 2.3. Carotid Artery Elastic Evaluation by ET

**Figure 3:**
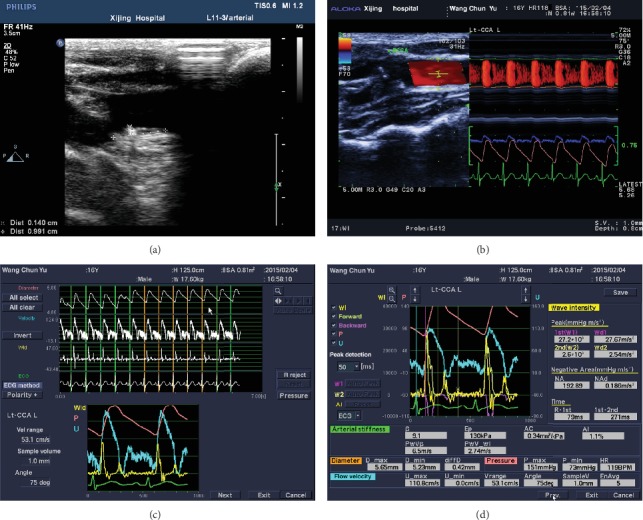
The carotid artery ultrasound evaluation of HGPS patients. (a) The borders of the plaque in the carotid artery in HGPS patients. (b) Left: screenshot of the RCCA tracing gate. Right: changes in vessel diameter. The Doppler flow and flow velocity were determined by ALOKA e-tracking. (c, d) B-mode, indicating the cursor locations and postanalysis screen.

All patients underwent carotid ultrasound scans with a 5-10 MHz transducer (Aloka F75, Hitachi, Japan). The measurements were performed in a standard manner, in which the patient was placed in the supine position, with a head elevation of up to 45° and a side tilt of 30° to the right and left [12]. The carotid artery stiffness was evaluated using the e-tracking option in a standard manner, and the parameters of the pressure-strain elastic modulus (Ep), artery stiffness parameters (*β*), arterial compliance (AC), augmentation index (AI), and pulse wave velocity (PWV*β*) were measured and analyzed (Figure 3).

### 2.4. Statistical Analysis

All statistical analyses were performed using the SPSS statistical software package (version 13.0; SPSS Inc., Chicago, IL, USA) and Microsoft Excel 2013 (Microsoft Corporation, Redmond, WA, USA). Data were presented as mean ± standard deviation ($\bar{x}\pm \text{SD}$). One-way ANOVA was used to compare the ultrasound parameters among HGPS children, age-matched healthy children, and older healthy volunteers. A *P* value of < 0.05 was considered statistically significant.

## 3. Results

### 3.1. Left Ventricle Remodeling and Ejection Fraction in HGPS Children

**Figure 4:**
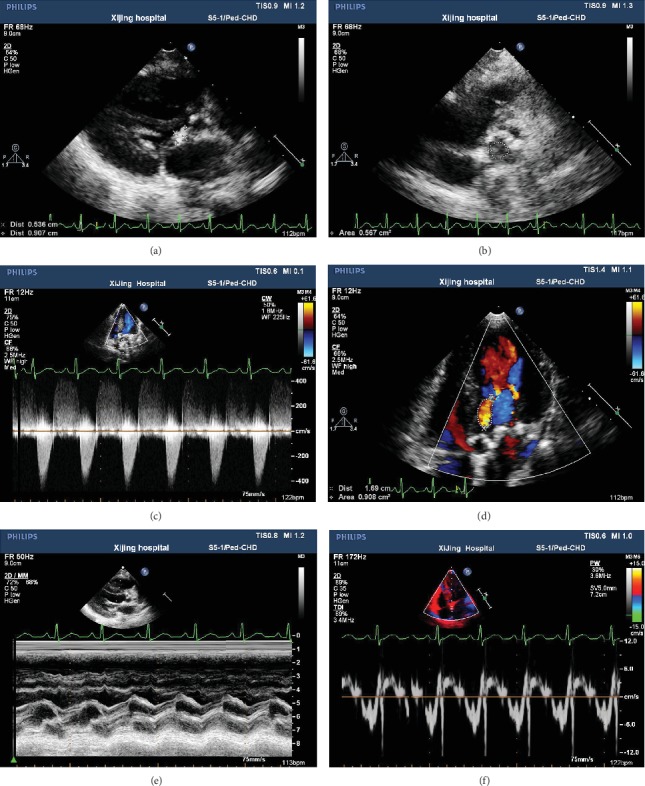
The conventional transthoracic echocardiographic examination of an elder HGPS patient (16 years old). (a) Aortic valve calcification.(b) The effective orifice area of systolic was 0.567 cm^2^. (c) The peak systolic velocity increase by color Doppler flow imaging. (d) The slight aortic valve regurgitation. (e) The left ventricular regional wall motion was normal by M-mode echocardiography. (f) The decrease in left ventricular diastole function by tissue Doppler imaging (TDI).

The echocardiographic data of the included participants are presented in Table 2. It was found that one child with HGPS had a calcified aortic valve (5 × 6 mm) with severe aortic stenosis (area of the aortic valve: 0.567 cm^2^; peak systolic gradient: 73 mmHg) and a remarkable intraventricular septum and LV hypertrophy (Figure 4). The other patient with HGPS had abnormal LV diastolic function (*E*′/*A*′ < 1). The LV end-systolic diameter (LVESD), end-diastolic volume (EDV), end-systolic volume (ESV), and mass were lower in HGPS children, when compared to age-matched children, while the LV end-diastolic diameter (LVEDD) was similar between HGPS and age-matched children. Furthermore, the ejection fraction in HGPS children was higher than that in older healthy volunteers but was similar with age-matched children (*P* > 0.05).

### 3.2. Three-Dimensional (3D) Strain Analysis

The results of the 3D strain index of participants in each group are presented in Table 3. The GLS of HGPS and age-matched children were higher, when compared to older healthy volunteers (*P* < 0.05) but was similar between HGPS and age-matched children (*P* > 0.05). However, there was no significant difference in the GCS of HGPS children, when compared to other controls (*P* > 0.05). Furthermore, there was no difference in the LV systolic twist or torsion among participants in the three groups (*P* > 0.05).

### 3.3. Left Ventricle Dyssynchrony Analysis

The LV mean peak time was lower in HGPS children, when compared to age-matched children and older healthy volunteers. Furthermore, the LV mean peak time was lower in age-matched healthy children, when compared to older healthy volunteers (*P* < 0.05). The SDI of HGPS (4.8 ± 1.8%) and age-matched (4.9 ± 1.8%) children were lower, when compared to older healthy volunteers (6.1 ± 2.9%), while no significant difference was detected for SDI between HGPS and age-matched children (*P* > 0.05).

### 3.4. Carotid Structural Changes

Cervical vascular abnormalities were found in five HGPS patients (83.3%), and the lesions became severer during the subsequent follow-ups. One of these patients had a typical manifestation of multiple carotid atherosclerotic plaques, while another patient presented with a curving internal carotid artery. The other three patients had thin arteries (two vertebral arteries and one internal carotid artery). Among older healthy volunteers, 10 volunteers had carotid atherosclerotic plaques (71.4%), while the remaining volunteers were normal. Furthermore, no carotid abnormalities were found in age-matched children.

### 3.5. Carotid Elasticity in HGPS Children

The index of the elasticity of the carotid in the three groups is presented in Table 4. The Ep, *β*, and PWV*β* of older healthy volunteers were higher, when compared with the other two groups, while AC was lower in older healthy volunteers (*P* < 0.05). Furthermore, there was no AI abnormality in these subjects. Moreover, no significant difference in the index of the elasticity was found between HGPS children and age-matched children.

## 4. Discussion

In the present study, it was found that HGPS children have impaired LV synchrony, when compared with normal children, although the LVEF was not significantly different. Furthermore, the structural abnormality of the carotid artery of HGPS children was similar with that of older healthy people, although the index of the elasticity appeared to be more favorable. These results suggest that in terms of the cardiovascular system of older healthy people, HGPS is different from natural aging.

HGPS was first described by Jonathan Hutchinson and Hastings Gilford in 1897 [13, 14]. Up to July 2015, a total of 125 children have been diagnosed with HGPS from 43 different countries [15]. The morbidity of HGPS is extremely rare, which is estimated to be approximately one in four million people [3]. Furthermore, the incidence of the disease is often underestimated due to the general lack of recognition of HGPS by clinicians. Both myocardial infarction and stroke have been accounted for more than 90% of patient deaths in HGPS [16]. Previous studies have indicated that vascular progerin is generated in young non-HGPS individuals, which significantly increases throughout life, strongly suggesting that progerin has a role in the cardiovascular aging of the general population [17]. However, the related structural and functional changes of the cardiovascular system in HGPS patients remain poorly understood. In the present study, it was found that the LV cavity of HGPS children was smaller than that of normal children, which may reflect the developmental delay and short stature of these patients. However, based on the results of the LVEF on 3D echocardiography and GLS, it was found that the LV function of HGPS patients was similar to that of normal children and was favorable, when compared with older healthy volunteers. Furthermore, similar changes in LV dyssynchrony were detected among these groups. These findings could have been possibly due to the relatively short duration of HGPS in these patients (16 years in one patient and between 1.5 and 4.0 years for the other patients). However, in the early stage of HGPS, serious cardiac changes did not occur. Interestingly, for the 16-year-old HGPS child who died after the one-year follow-up, GLS was found to be much lower than that in other patients with HGPS, which is consistent with previous findings, in which GLS could serve as a prognostic index in patients with various cardiac diseases [18], including heart failure [19].

With regard to the carotid artery, these present findings revealed that one elder case of HGPS appeared to have multiple atherosclerotic plaques, while the remaining cases of HGPS only presented with carotid intima-media thickness, abnormal diameter, spectrum form and blood flow velocity, or even normal carotid artery. These findings were consistent with the results of a previous study, which revealed that the vasculature of HGPS patients presented with excessive calcification, lipid accumulation, vessel wall thickening, and fibrosis due to smooth muscle cell (SMC) malfunction [17]. These changes may be associated with the high level of progerin accumulation. By gradually raising the levels of progerin in human cells, Chojnowski et al. found that DNA damage and cell senescence only occurs when the amount of progerin in a cell exceeds a particular threshold [20]. For elasticity changes, these were similar in HGPS and normal children and were favorable, when compared to older healthy volunteers. Meanwhile, it was found that arterial elasticity decreased, while the stiffness of the arteries remarkably increased in HGPS with aging. These present findings are consistent with previous studies, which also revealed the accelerated vascular stiffening in patients with HGPS, as reflected by the impaired carotid-femoral pulse wave velocity [6]. These present results reveal that the elasticity initially appeared to be abnormal with aging. This could be considered an important risk factor for predicting HGPS cardiovascular aging, which may be correlated to vascular adventitial fibrosis and the replacement of vascular SMCs through the extracellular matrix or fibrous tissue in HGPS [21–23], finally leading to arteriosclerosis.

## 5. Conclusion

To our best knowledge, the present study is the first to report the comparison of the functional and structural characteristics of the cardiovascular system among patients with HGPS, normal children, and older healthy volunteers using advanced ultrasonography. Although the present study is limited by the small sample size of the included HGPS patients, these results indicate that changes in the cardiovascular system in HGPS are different from natural aging. The results of the present study may provide an additional understanding of the potential vulnerability of children with HGPS to cardiovascular diseases.

## Figures and Tables

**Table 1:** Clinical characteristics of HGPS (first consultation).

Age (years)	Weight (kg)	Height (cm)	Blood pressure (mmHg)	Head circumference (cm)
1.8	8.2	75	88/62	45.9
2.8	7.7	78	94/64	48.5
4	7.9	77	95/72	44.5
7	9.5	93	89/78	44.3
8	8.8	96	92/70	45.5
15	17.6	125	102/80	48.0
17	15.9	113.5	110/75	47.3

**Table 2:** Echocardiographic data of the included participants ($\bar{x}\pm \text{SD}$).

	42	LVEDD (mm)	LVESD (mm)	LVEDV (mL)	LVESV (mL)	LVEF (%)	Mass (g)
HGPS children	7	34.7 ± 7.6^b^	16.7 ± 2.8^ab^	21.0 ± 7.6^ab^	8.1 ± 3.9^ab^	62.4 ± 8.8^b^	31.9 ± 10.5^ab^
Age-matched healthy children	21	32.8 ± 3.2^b^	21.95 ± 2.3^b^	42.9 ± 14.8^b^	16.0 ± 6.1^b^	62.7 ± 5.9^b^	62.8 ± 20.7^b^
Older healthy volunteers	14	44.3 ± 3.1^a^	30 ± 2.3^a^	76.9 ± 24.1^a^	36.7 ± 14.1^a^	53.4 ± 13.2^a^	119.6 ± 36.5^a^
F		48.313	101.227	74.772	46.059	13.641	85.529
*P*		0.000	0.000	0.000	0.000	0.000	0.000

Statistics were performed using one-way ANOVA, followed by Fisher's post hoc test. Data were presented as mean ± standard deviation (SD). ^a^*P* < 0.05, when compared with age-matched healthy children; ^b^*P* < 0.05, when compared with older healthy volunteers. LVEDD: left ventricular end-diastolic diameter; LVESD: left ventricular end-systolic diameter; LVEDV: left ventricular end-diastolic volume; LVESV: left ventricular end-systolic volume; EF: ejection fraction.

**Table 3:** Echocardiographic parameters of the strain and dyssynchrony.

		GLS%	GCS%	Twist (°)	Torsion°	Mean peak time (ms)	SDI%
HGPS children	7	−20.6 ± 4.0^b^	−27.8 ± 6.0	7.9 ± 9.6	1.9 ± 2.6	249.7 ± 33.1^ab^	4.9 ± 1.8
Age-matched healthy children	21	−21.2 ± 3.2^b^	−28.3 ± 5.2^b^	11.7 ± 7.6	2.0 ± 1.2	290.2 ± 48.5^b^	4.8 ± 1.8^b^
Older healthy volunteers	14	−17.3 ± 4.4^a^	−24.2 ± 8.5^a^	11.5 ± 10.9	1.9 ± 1.2	309.3 ± 40.3^a^	6.1 ± 2.9^a^
F		13.978	5.284	1.277	0.020	11.226	3.885
*P*		0.000	0.006	0.282	0.980	0.000	0.023

^a^ *P* < 0.05, when compared with age-matched healthy children; ^b^*P* < 0.05, when compared with older healthy volunteers. GLS: global longitudinal strain; GCS: global circumferential strain.

**Table 4:** Echocardiographic parameters of carotid elasticity.

	Ep	*β*	AC	AI	PWV*β*
HGPS children	36.20 ± 13.2^b^	3.14 ± 1.22^b^	1.28 ± 1.55	12.38 ± 10.69	3.68 ± 0.69^b^
Age-matched healthy children	32.7 ± 5.85^b^	3.3 ± 0.59^b^	1.57 ± 0.27^b^	9.19 ± 13.66	3.53 ± 0.31^b^
Older healthy volunteers	125.2 ± 66.60^a^	9.89 ± 5.24^a^	0.84 ± 0.42^a^	12.97 ± 8.35	6.48 ± 1.70^a^
*F* value	24.889	20.616	8.536	0.611	37.530
*P* value	0.000	0.000	0.001	0.547	0.000

^a^ *P* < 0.05, when compared with age-matched healthy children; ^b^*P* < 0.05, when compared with older healthy volunteers.

## Data Availability

The datasets generated and analyzed during the current study are available from the corresponding author on reasonable request.
